# Identification and characterization of endogenous biomarkers for hepatic vectorial transport (OATP1B3-P-gp) function using metabolomics with serum pharmacology

**DOI:** 10.1007/s00726-023-03363-5

**Published:** 2024-02-06

**Authors:** Yong-wen Jin, Yan-rong Ma, Ming-kang Zhang, Wen-bin Xia, Pei Yuan, Bo-xia Li, Yu-hui Wei, Xin-an Wu

**Affiliations:** 1 Department of Pharmacy, The First Hospital of Lanzhou University, Lanzhou, 730000 China; 2 The First Clinical Medical College, Lanzhou University, Lanzhou, 730000 China; 3 School of Pharmacy, Lanzhou University, Lanzhou, China

**Keywords:** OATP1B3, P-gp, Endogenous biomarker, Metabolomics

## Abstract

The organic anion-transporting polypeptide 1B3 and P-glycoprotein (P-gp) provide efficient directional transport (OATP1B3-P-gp) from the blood to the bile that serves as a key determinant of hepatic disposition of the drug. Unfortunately, there is still a lack of effective means to evaluate the disposal ability mediated by transporters. The present study was designed to identify a suitable endogenous biomarker for the assessment of OATP1B3-P-gp function in the liver. We established stably transfected HEK293T-OATP1B3 and HEK293T-P-gp cell lines. Results showed that azelaic acid (AzA) was an endogenous substrate for OATP1B3 and P-gp using serum pharmacology combined with metabolomics. There is a good correlation between the serum concentration of AzA and probe drugs of rOATP1B3 and rP-gp when rats were treated with their inhibitors. Importantly, after 5-fluorouracil-induced rat liver injury, the relative mRNA level and expression of rOATP1B3 and rP-gp were markedly down-regulated in the liver, and the serum concentration of AzA was significantly increased. These observations suggest that AzA is an endogenous substrate of both OATP1B3 and P-gp, and may serve as a potential endogenous biomarker for the assessment of the function of OATP1B3-P-gp for the prediction of changes in the pharmacokinetics of drugs transported by OATP1B3-P-gp in liver disease states.

**Supplementary Information:**

The online version contains supplementary material available at 10.1007/s00726-023-03363-5.

## Introduction

The liver is mainly responsible for the conversion of drugs and other xenobiotic compounds to inactive forms, many of which are eliminated in the bile. Currently, biological markers of “liver function” (such as ALT, AST and ALP) are used as markers for drug dosage adjustments. This measure of “liver function” does not necessarily correlate with changes in drug disposition, but rather indicates hepatocyte damage. Specific markers related to the hepatic disposition of drugs are needed to guide drug dosage adjustments (Verbeeck 2008). Hepatocytes express a variety of drug transporters in the blood-facing sinusoidal and bile-facing canalicular membranes, forming efficient directional transport from the blood circulation to the bile (Maeda and Sugiyama 2008). Illness, genetic variation and drug–drug interactions result in pharmacokinetic differences for the substrates of these drug transporters (Hirota et al. 2020; Yee et al. 2018). Therefore, predicting transporter function in the liver is important for predicting the pharmacokinetic properties of new drug candidates in humans and their pharmacological effectiveness and adverse reactions. The US Food and Drug Administration (FDA) and other regulatory agencies have recommended the use of suitable markers to assess the function of transporters such as P-gp and OATP1B3 (Amawi et al. 2019; Sudsakorn et al. 2020; Yoshida et al. 2017; Zhang et al. 2009).

Some endogenous substrates of drug transporters have emerged as potential biomarkers to assess differences in drug transporter function (Müller et al. 2018). Organic anion transporting polypeptides (OATP)1B1 and OATP1B3 are liver-specific transport proteins that facilitate the uptake of a variety of clinically important drugs and endogenous substrates such as bilirubin, thyroid hormones, bile acids and their conjugates (Barnett et al. 2019). OATP1B1 and OATP1B3 have different zonal expression profiles and substrate specificity in the liver, among which OATP1B3 is expressed primarily around the central vein of hepatic lobules (König et al. 2000) and mediates the uptake of digoxin, glibenclamide, glipizide, etc. (Chen et al. 2018). Due to the overlap and specificity of the substrates of OATP1B1 and OATP1B3, it is particularly important to specifically evaluate OATP1B1 or OATP1B3. This study aimed to identify and characterize an endogenous biomarker for specific evaluation of OATP1B3 function. CCK-8 is mainly transported by OATP1B3 and can be used as a probe substrate (Alam et al. 2017). However, CCK-8 is rapidly degraded in the blood and its half-life is about 3 min, which is not suitable as a functional marker in vivo. P-gp is abundant in the bile canalicular membranes of hepatocytes and functions to transport substrate drugs (i.e., digoxin, imatinib, doxorubicin, fexofenadine, ouabain) and other compounds from the hepatocyte into the bile (Liu 2019). An endogenous substrate for P-gp that can serve as a functional marker has not yet been identified. Substrate drugs of OATP1B3 and P-gp have a large overlap, thereby mediating the vectorial transport of the same drug from the blood to bile. Thus, it is necessary to find endogenous biomarkers to evaluate the function of the OATP1B3 and P-gp vectorial transport (OATP1B3-P-gp) to predict changes in the pharmacokinetics of drugs transported through OATP1B3-P-gp in liver disease states.

Metabolomics, which serves as a powerful platform focused on the comprehensive profiling of small metabolites, has provided a promising approach to biomarker discovery (Cao et al. 2022). We utilized a serum pharmacology method as our research approach. Stably transfected cell lines were incubated in culture media containing human serum powder from patients with liver cirrhosis. Metabolomics was used to discover the appropriate endogenous biomarker for the assessment of the function of OATP1B3-P-gp in the liver.

## Methods

### Chemicals

Rifampicin (RIF), verapamil (Ver), ritonavir (RTV), pravastatin (PV), 5-fluorouracil and fexofenadine (FEX) were purchased from Shanghai Macklin Co., Ltd (Shanghai, China), Tetradecanedioate (TDA), hexadecanedioate (HAD), 1-methylnicotinamide, pyridoxine, N-acetyl-L-phenylalanine, N-acetylvaline and azelaic acid (AZA) were purchased from Sigma-Aldrich (St Louis, MO, USA). L-serine-O-sulfate was purchased from Toronto Research Chemicals (Toronto, Canada). All primers for the real-time quantitative polymerase chain reaction (PCR) detecting system (qPCR) or quantitative real-time polymerase chain reaction (qRT-PCR) were synthesized by Sunbiotech Co., Ltd. (Beijing, China). Anti-P-gp was purchased from Abcam (ab170904, rat, USA). Anti-Oatp1b2 was purchased from Santa Cruz Biotechnology (sc-376904, rat, USA). Anti-β-actin was purchased from Affinity (AF7018, rat, China). Methanol and acetonitrile were high-performance liquid chromatography (HPLC)-grade (Fisher Scientific, NJ, USA). All other reagents and solvents were of analytical grade and were commercially available.

### Animals

Male SD rats aged 3 months, weighing 200–220 g, were obtained from the Experimental Animal Center of Lanzhou Institute of Biological Products Co., Ltd (Lanzhou, China). Rats were housed in plastic cages and maintained at 25 °C under a 12 h alternating light–dark cycle with free access to food and water. All studies were granted by the Research Ethics Committee of First Hospital of Lanzhou University (Approval NO: LDYYLL-2022-29).

### Cell culture

Empty vector (mock) transfected and human OATP1B1, OATP1B3 and P-gp transfected human embryonic kidney 293 cell line (HEK293T) or canine kidney cell (MDCK), were cultured using high-glucose DMEM supplemented with 10% FBS, 100 U/mL penicillin and 100 µg/ml streptomycin at 37 °C and 5% CO_2_. All cells were sub-cultured twice a week using trypsin (0.05%)-EDTA (0.02%) solution.

### Cellular uptake of substrates and metabolites

HEK293T-OATP1B3, MDCK-OATP1B1 and HEK293T-P-gp cells were transfected with the GV lentivirus vectors using the Transfection Reagent Kit (Genechem Co., Ltd). HEK293T/MDCK cells (3–5 × 10^4^ cells/mL, 2 mL/well) were inoculated into 6-well plates. After 24 h of culture, GV lentivirus vectors (5–8 × 10^7^ titer) and transfection solution were added for further incubation of 16–24 h. Then the transfection solution was replaced with a medium to continue the culture. After 72 h, puromycin (4–8 μg/mL) was added for 48 h to obtain the stably transfected cell lines. Laser scanning confocal microscopy (LSCM, LSM710, Zeis) was used to observe the transfection efficiency. An initial amount of 5 × 10^5^ cells/well was cultured in a glass bottom dish. After 12 h of culture, cells were stained with DAPI. qPCR was used to quantify the relative expression of messenger RNA (mRNA) of OATP1B1, OATP1B3 and P-gp in transfected cell lines. Sequences for the primers are listed in Table s1. Transport activity of OATP1B1, OATP1B3 and P-gp in stably transfected cells was verified by analyzing the uptake of the substrate PV (100 µM), PV (100 µM) and FEX (100 µM), respectively. The target gene contained a 3 kDa tagged protein sequence; the detection of the tagged protein by WB was used to investigate the expression of the target gene.

Cellular uptake in the HEK293T-Mock, MDCK-Mock, HEK293T-OATP1B3, MDCK-OATP1B1 and HEK293T-P-gp cell lines was analyzed as previously described (Taghikhani et al. 2017). Cells were cultured in poly-L-lysine-coated 12-well plates at an initial density of 5 × 10^5^ cells/well at 37 °C for 24 h before induction by replacing the medium with fresh medium containing 10 mM of sodium butyrate. After 24 h of incubation, the cells were used for the following cellular uptake studies.

Uptake of substrates or metabolites at 37 °C and 4 °C: The medium was replaced with pre-warmed (37 °C) Krebs–Henseleit buffer containing 100 μM substrates or metabolites to uptake for 20 min. Cells were placed in a 4 °C environment for 20 min, then buffer containing pre-cooled (4 °C) Krebs–Henseleit buffer containing 100 μM metabolites was added at 4 °C to uptake for 20 min.

Inhibition of uptake and efflux of AZA: The medium was replaced with pre-warmed (37 °C) Krebs–Henseleit buffer containing 100 μM AZA and 10 or 50 μM RIF, Ver and RTV to uptake for 20 min.

Transport kinetics of AZA: uptake was initiated by adding pre-warmed (37 °C) Krebs–Henseleit buffer containing 100 μM AZA and performed for 2.5 min, 5 min, 10 min, 20 min and 30 min, after which linear uptake times were determined. To obtain the uptake affinity of AZA, liner uptake time was selected as a representative of uptake rate and used to measure concentration-dependent (50, 100, 200, 400, 800, 1000 and 1500 µM) uptake in HEK293T-Mock and HEK293T-OATP1B3 cells, and the Km of AZA taken up by OATP1B3 were calculated according to the Michaelis–Menten equation.

Uptake was terminated by adding 1 mL/well of pre-cooled (4 °C) Krebs–Henseleit buffer, followed by two washes, and lysed using 200 µL of deionized water. The protein concentration of the lysate was measured by BCA Kit. The concentration of substrate was analyzed by Liquid Chromatography-Mass Spectrometry/Mass Spectrometry (LC–MS/MS).

### Western blotting

Liver tissues were homogenized in RIPA buffer (containing serine protease inhibitor PMSF) and then centrifuged at 14,000 g for 10 min at 4 ℃. Protein concentration was determined by BCA protein assay kit, and the supernatant was subjected to Western blot (WB) analysis. Proteins were separated by SDS-PAGE electrophoresis and transferred to a methanol-activated PVDF membrane. PVDF membranes were blocked with 5% skim milk (dissolved in TBS-T) for 1 h at room temperature and then incubated with Anti-Oatp1b2, Anti-P-gp, and Anti-β-actin for 2 h. The signal was then incubated with HRP-conjugated secondary antibody for 1 h and detected with enhanced chemiluminescence (ECL) solution. Automatic Chemiluminescence/Fluorescence Image Analysis System (Tanon 4600 Series, China) was used for chemiluminescence color development, followed by grayscale value counting using Image J software.

### Study design

The first step was to establish stably transfected cells. The open reading frame of SLCO1B1 (NM_006446), SLCO1B3 (NM_019844) and ABCB1 (NM_000927) was amplified by PCR and cloned into the expression carrier GV492 (Shanghai Genechem Co., Ltd., Shanghai, China). GV492 carries an anti-puromycin gene, GFP protein gene-tagged and the target gene (SLCO1B1, SLCO1B3 or ABCB1).

The second set of experiments was to identify endogenous substrates for OATP1B3 and P-gp by serum pharmacology combined with metabolomics. This study was conducted in 30 cirrhosis patients and 30 healthy volunteers. Medical records/serum samples obtained in previous clinical diagnosis and treatment were used in the research granted by the Research Ethics Committee of First Hospital of Lanzhou University (Approval NO: LDYYLL-2022-29). The mixture of serum of cirrhosis patients was spin-dried at 1000 × g at 45 °C, and the same volume of Krebs–Henseleit buffer was added to dry serum powder as endogenous substrate sources (equivalent to the serum concentration in humans) to dissolve the sample and the filtrate served as endogenous compounds source. The Krebs–Henseleit buffer containing the endogenous substance was used for uptake studies in HEK293T-Mock and HEK293T-OATP1B3 cells as described in the “Cellular uptake of substrates and metabolites” section. Cell lysates were used for untargeted metabolomics which was conducted by Biotree Biomedical Technology. The Metabolon analytic platform incorporates high-performance liquid chromatography/tandem mass spectrometry injections (Q Exactive Orbitrap, Thermo Fisher Scientific, USA); multivariate analysis was applied for data statistics.

The third series of experiments utilized the differential metabolites to identify a substrate of OATP1B3 and P-gp but not of OATP1B1. We identified and purchased differential metabolites that are poorly or un-metabolized in the liver. These differential metabolites were used for the uptake studies in HEK293T-OATP1B3, MDCK-OATP1B1, HEK293T-P-gp, HEK293T-Mock and MDCK-Mock cell lines as described in the “Cellular uptake of substrates and metabolites” section.

The fourth series of experiments investigated the effect of RIF or Ver on the serum concentration of AzA, PV and FEX. In the first experiment, rats were divided into four groups: RIF Control, RIF, Ver Control and Ver group rats (n = 6 each). These groups were treated orally with 0.5% CMC–Na, RIF (30 mg/kg in 0.5% CMC–Na), 0.5% CMC–Na and Ver (50 mg/kg in 0.5% CMC–Na), respectively. Blood and liver tissue samples were collected 2 h after a single administration, and AzA was measured by LC-MS/MS. In the second experiment, rats were divided into four groups: RIF Control, RIF, Ver Control and Ver group rats (n = 5 each) were treated orally with 0.5% CMC–Na, RIF (30 mg/kg in 0.5% CMC–Na), 0.5% CMC–Na and Ver (50 mg/kg in 0.5% CMC–Na) respectively for 7 d consecutively. Blood and liver tissue samples were collected 2 h after the last administration and AzA was measured by LC-MS/MS. In the third experiment, rats were divided into four groups: Control, PV+RIF (30), PV+RIF (60) and PV+RIF (120) group (n = 5 each) were treated orally with PV (20 mg/kg), PV (20 mg/kg) + RIF (30 mg/kg), PV (20 mg/kg) + RIF (60 mg/kg) and PV (20 mg/kg) +RIF (120 mg/kg), respectively. Blood and hepatic tissue samples were collected 2 h after a single administration, and AzA and PV were measured by LC-MS/MS. In the fourth experiment, rats were divided into four groups: Control, FEX+Ver (25), FEX+Ver (50) and FEX+Ver (100) group (n = 5 each) were treated orally with FEX 20 mg/kg, FEX (20 mg/kg) + Ver (20 mg/kg), FEX (20 mg/kg) + Ver (60 mg/kg) and FEX (20 mg/kg) + Ver (100 mg/kg), respectively. Blood and hepatic tissue samples were collected 2 h after a single administration and AzA and FEX were measured by LC-MS/MS.

The fifth series of experiments was to evaluate the influence of liver injury on the levels of the endogenous biomarker AzA. In the first study, a rat model of liver injury was established by intraperitoneal injection (i.p.) of 5-fluorouracil. Rats were divided into two groups: Control (i.p with corn oil) and 5-fluorouracil (i.p, 20 mg/kg) for 7 d consecutively. Blood and hepatic tissue samples were collected at 2 h after the final 5-fluorouracil administration, and AzA was measured by LC–MS/MS. AST, ALT, ALP and ALB were determined by a fully automated chemistry analyzer (OLYMPLLS AU2700, Olympus Co., Tokyo, Japan). Liver tissue was harvested, and fixed in 10% neutral-buffered formalin for hematoxylin and eosin (H&E) staining. WB was used to analyze the change in expression of Oatp1b2 and P-gp in rat liver. The total liver RNA of rats was extracted with Eastep^®^ Super Total RNA Extraction Kit (Promega, LS1040). After determining the RNA concentration (NanodropPharmaceutics 2000, ThermoFisher) of the samples and diluting to the same concentration (50–150 ng/uL), the extracted RNA was reverse transcribed into cDNA using RevertAid First Strand cDNA Synthesis Kit (Thermo Scientific, K1622). qRT-PCR (BIO-RAD, CFX96) was used to measure the relative mRNA expression of the Oatp1b2, P-gp and Oatp1a4 using the UltraSYBR Mixture (Low ROX) (CWBIO, CW2601). β-actin was the internal reference. The 2^−△△Ct^ method was used to calculate the relative mRNA expression. In the second experiment, rats were divided into six groups: RIF Control, RIF, Ver Control, Ver group, 5-fluorouracil Control (i.p with corn oil) and 5-fluorouracil (n = 6 each). Rats and were treated orally with 0.5% CMC–Na, RIF (30 mg/kg in 0.5% CMC–Na), 0.5% CMC–Na, Ver (50 mg/kg in 0.5% CMC–Na), corn oil (i.p), and 5-fluorouracil (i.p, 20 mg/kg) respectively for 7 d consecutively. 2 h after the last dose, rats were anesthetized with isoflurane and fixed. The abdomen was opened approximately 2 cm below the xiphoid process, and the bile duct was isolated for cannulation to bile duct. Bile samples were collected for 1 h. All samples were weighed, and frozen at − 80 °C. AzA was measured by LC–MS/MS. In the third study, the serum level of AzA in thirty healthy volunteers and thirty cirrhosis patients was determined by LC–MS/MS.

### Sample preparation and LC–MS/MS analysis

Cell lysates (50 μL) were added to 50 μL of the internal standard (IS) with 50 μL methanol for deproteinization. Following vortex mixing and centrifugation at 14,000 g for 10 min, the upper layer was analyzed. Serum samples were centrifuged at 14,000 g for 10 min. The supernatant serum layer (50 μL) and bile (50 μL) were mixed with 50 μL of the (IS) and 100 μL methanol for deproteinization. Tissues were weighed and homogenized (0.2 g/400 μL saline), and then centrifuged at 14,000 g for 10 min, after which supernatant (50 μL) was added to 50 μL of the IS with 100 μL methanol. Following vortex mixing and centrifugation at 14,000 g for 10 min, the upper layer was analyzed with an Agilent 1260 HPLC coupled to an Agilent 6460 Triple Quadrupole mass spectrometer equipped with ESI or an APCI interface. Quantification was performed using multiple reaction monitoring (MRM). The calibration curves were linear over the concentration range with a coefficient (r) of 0.99.

### Data analysis and statistical analysis

Statistical analysis was computed using IBM SPSS Statistics 22 software. Data are expressed as mean ± SD. Statistically significant differences between the two sets were compared using a one-way analysis of variance. In all statistical analyses, *P* < 0.05, *P* < 0.01, or *P* < 0.001 was considered to indicate a statistically significant result.

## Results

### Establishment of stably transfected cell lines

**Fig. 1:**
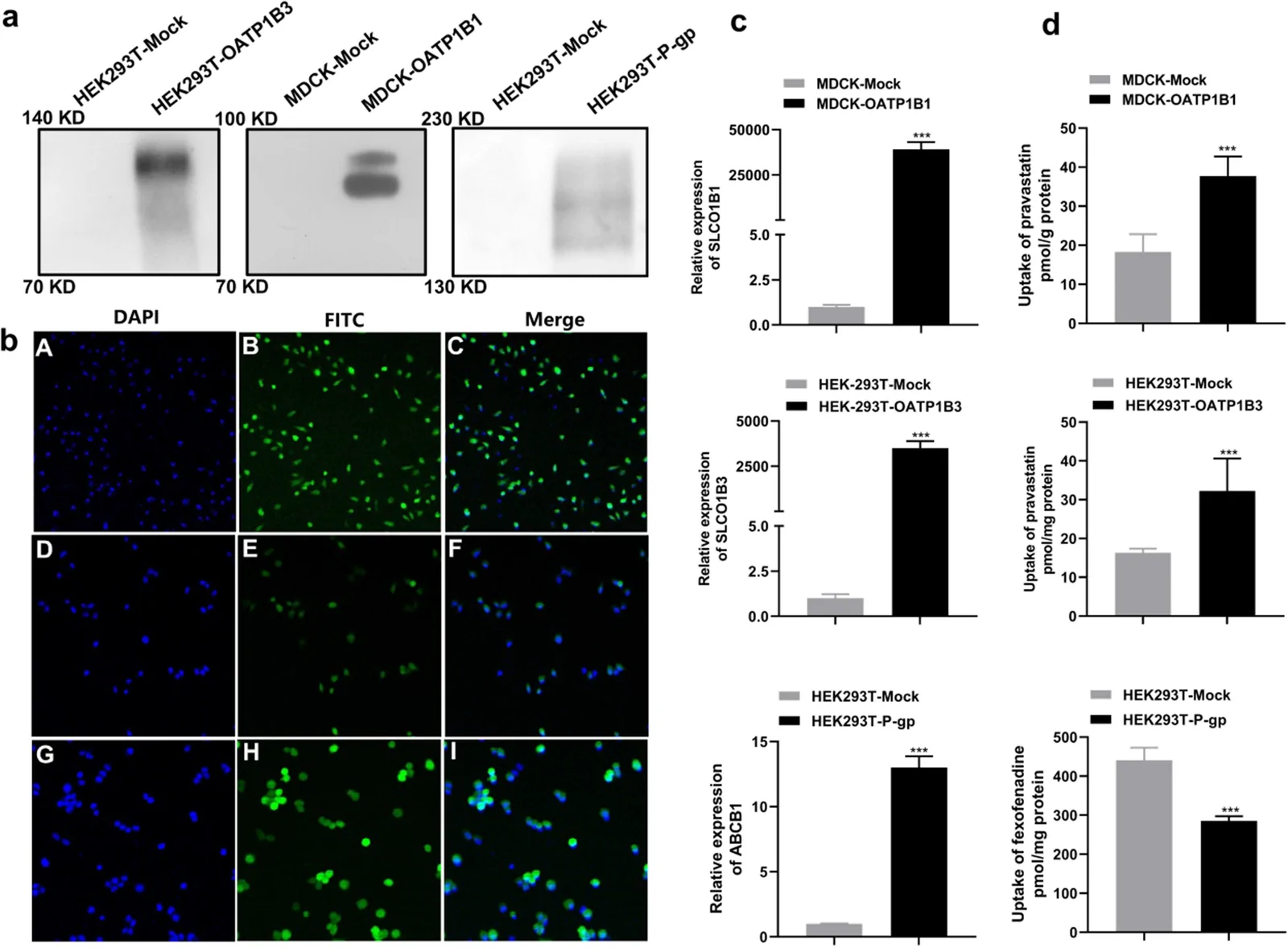
Stably transfected cell lines. **a**: The detection of tagged protein by western blot; **b**: the results of laser scanning confocal microscopy of MDCK-OATP1B1 (A, B, C), HEK293T-OATP1B3 (D, E, F) and HEK293T-P-gp (G, H, I) cells; blue: stained with 4ʹ,6-diamidino-2-phenylindole (DAPI), green: fluorescein isothiocyanate (FITC) flag. **c** and **d**: relative expression of messenger RNA and transport activity of OATP1B1, OATP1B3 and P-gp in transfected cell lines. Data are expressed as mean ± SD. (n = 6). ***P < 0.001 vs. MDCK-Mock or HEK293T-Mock, respectively

Lentiviruses are powerful tools for gene delivery and have been widely used for the long-term overexpression of miRNAs in nearly all cell types. In the present study lentiviruses carrying the gene fragments of SLCO1B1, SLCO1B3 and ABCB1 were used to infect HEK293T and MDCK cells. The WB results show that a band with a molecular mass close to 80 kDa and 100 kDa was detected in MDCK-OATP1B1 and HEK293T-OATP1B3 cells, respectively. For P-gp, a band with a molecular mass close to 170 kDa was detected in HEK293T-P-gp cells (Fig. 1a). Enhanced fluorescent protein (EGFP) gene was significantly expressed in the MDCK-OATP1B1, HEK293T-OATP1B3 and HEK293T-P-gp cell lines as observed by LSCM (Fig. 1b). Analysis of mRNA expression by qPCR, and quantified by comparison to the expression of the housekeeping gene β-actin showed that SLCO1B1, SLCO1B3 and ABCB1 mRNA expression was increased by 39,101-fold, 3499-fold and 14-fold in MDCK-OATP1B1, HEK293T-OATP1B3 and HEK293T-P-gp cells, respectively (Fig. 1c). To measure OATP1B1, OATP1B3 and P-gp transport activity in the transfected cells, the uptake of OATP1B1, OATP1B3 and P-gp substrates PV, PV and FEX, respectively, was measured. The amount of PV in the MDCK-OATP1B1 and HEK293T-OATP1B3 cells was increased by 2.1-fold and 2.0-fold, respectively, compared to the corresponding HEK293T/MDCK-Mock cells. The amount of FEX in the HEK293T-P-gp cells was decreased by 1.5-fold compared to HEK293T-Mock (Fig. 1d). TDA and HAD were used for further confirmation of the specific transport activity of OATP1B1 and OATP1B3. TDA (10 μM) and HAD (10 μM) were transported by MDCK-OATP1B1 cells but not by HEK293T-OATP1B3 cells (Fig. S1).

### Participant demographics

Thirty healthy participants and thirty cirrhosis patients were enrolled in this study. The range for age, and serum levels of aspartate aminotransferase (AST), alanine aminotransferase (ALT), alkaline phosphatase (ALP), total bilirubin (TBIL), direct bilirubin (DBIL), indirect bilirubin (IBIL), glutamyl transferase (GGT), total bile acids (TBA) were shown in Table 1. All the relevant biochemical indicators were significantly increased in cirrhosis patients compared to the healthy participants (*P* < 0.001). The healthy participants did not manifest any abnormality by medical examination or blood or biochemical tests at baseline.

**Table 1:** Basic characteristics of the included patients

Group	Age (years)	Sex (male)	AST	ALT	ALP	TBIL	DBIL	IBIL	GGT	TBA
Normal	43.9 ± 10.7	48.6%	25.0 ± 5.8	30.5 ± 14.3	76.1 ± 17.4	14.7 ± 4.7	2.7 ± 0.8	12.0 ± 3.9	18.74 ± 9.2	12.6 ± 19.2
Patients	46.0 ± 10.8	53.3%	84.5 ± 56.2^a^	59.9 ± 46.7^a^	160.8 ± 95.1^a^	60.8 ± 47.5^a^	30.5 ± 34.5^a^	34.7 ± 23.3^a^	116.1 ± 81.3^a^	51.1 ± 48.0^a^

The results are shown as mean ± SD, n = 30

*AST* aspartate aminotransferase, *ALT* alanine aminotransferase, *ALP* alkaline phosphatase, *TBIL* total bilirubin, *DBIL* direct bilirubin, *IBIL* indirect bilirubin, *GGT* glutamyl transferase, *TBA* total bile acids

^a^p < 0.001 vs. Normal

### Untargeted metabolomics screening for endogenous substrates of OATP1B3

**Fig. 2:**
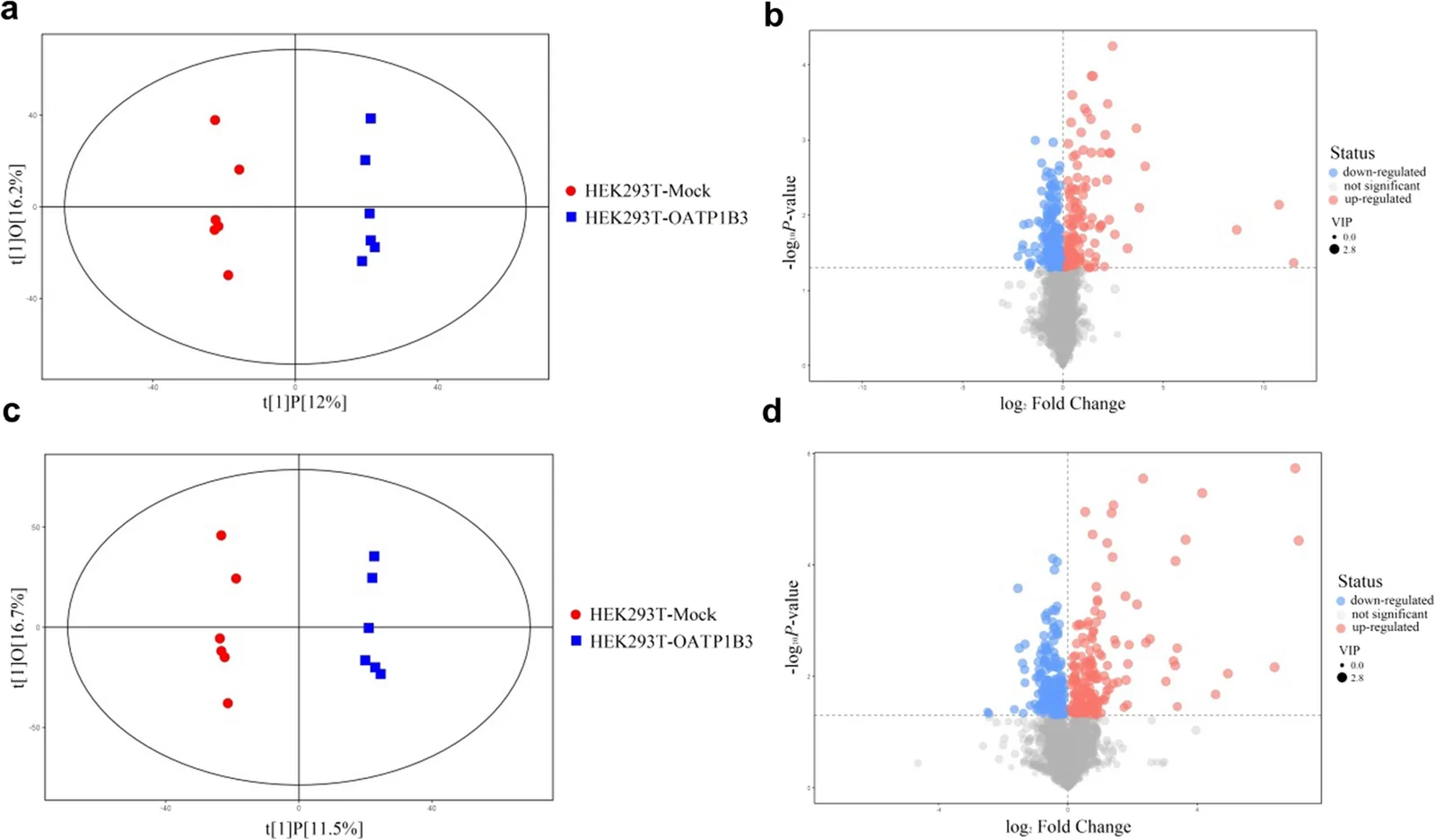
Serum derived-OATP1B3-overexpressing cells metabolite uptake profiles in OPLS-DA and volcano plots; positive ion modes (**a**, **b**) and negative ion modes (**c**, **d**). OPLS-DA: orthogonal partial least squares discriminant analysis

We present an untargeted approach using LC–MS/MS to illustrate the endogenous substrates of OATP1B3 after transport experiments in Krebs–Henseleit buffer with HEK293T-Mock and HEK293T-OATP1B3 cells. The orthogonal partial least squares discriminant analysis (OPLS-DA) revealed a clear discrimination between HEK293T-Mock and HEK293T-OATP1B3 cells (Fig. 2a and b). We identified nineteen and forty-three significantly increased metabolites in the negative and positive ion modes of mass spectrometry, respectively (Fig. 2c and d). Significantly increased metabolites, 1-methylnicotinamide, pyridoxine, L-serine-O-sulfate, AzA, N-acetylornithine, and N-acetyl-L-phenylalanine, showing VIP greater than 1.5 and p-value lower than 0.05 are selected for further investigated in vitro and *vivo* (Table 2).

**Table 2:** List of increased differential metabolites (n = 6)

Name	MEAN HEK293T-OATP1B3	MEAN HEK293T-Mock	VIP	*p* value
1-Methylnicotinamide	0.051793105	0.046639907	2.197071492	0.006405209
Pyridoxine	0.209476199	0.058824074	2.339019443	0.032385825
L-Serine-O-sulfate	0.003277663	0.002642044	1.755664297	0.04570739
Azelaic acid	0.00038101	0.000232587	2.071560976	0.005113825
N-Acetylornithine	0.00108073	0.00058759	2.416609223	0.001055526
N-Acetyl-L-phenylalanine	0.00041701	0.00036344	2.161418253	0.009797162

MEAN OATP1B3/Mock: relative quantitative value of the metabolite in HEK293T-OATP1B3 or -Mock groups within the set of comparisons; VIP: the variable projected importance of metabolite obtained from the OPLS-DA for this group of comparisons; *p* value: the *p* value obtained from the t-test for the comparison of this metabolite in the group. OPLS-DA: orthogonal partial least squares discriminant analysis

### Validation of endogenous substrates in vitro

**Fig. 3:**
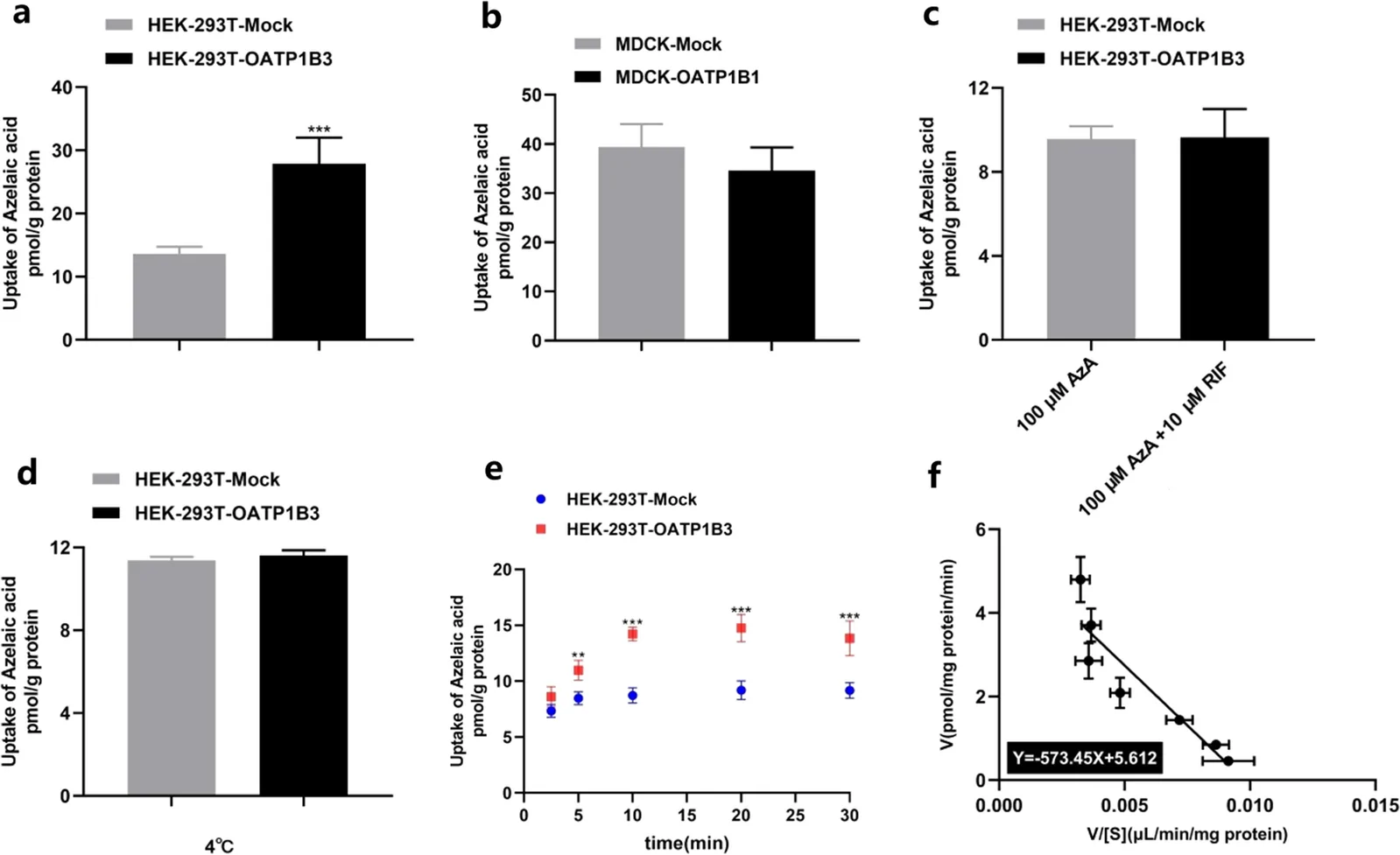
After 20 min of cellular uptake, AzA (100 μM) was taken up by OATP1B3 over-expressing cells (**a**) but not by OATP1B1 cells (**b**), and the transport activity was inhibited by RIF (10 μM) (**c**) and at 4 °C (**d**). Time-dependent and concentration-dependent (10 min) uptake was quantified by measuring the amount of AzA in the cells at different times (2.5, 5, 10, 20, 30 min) (**e**) and concentrations (50, 100, 200, 400, 800, 1000 and 1500 µM) (**f**). The Km was calculated according to the Michaelis–Menten equation. Data are expressed as mean ± SD. (n = 6); **P < 0.01, ***P < 0.001 vs. MDCK-Mock or HEK293T-Mock, respectively; *AzA* Azelaic acid, *RIF* Rifampicin

**Fig. 4:**
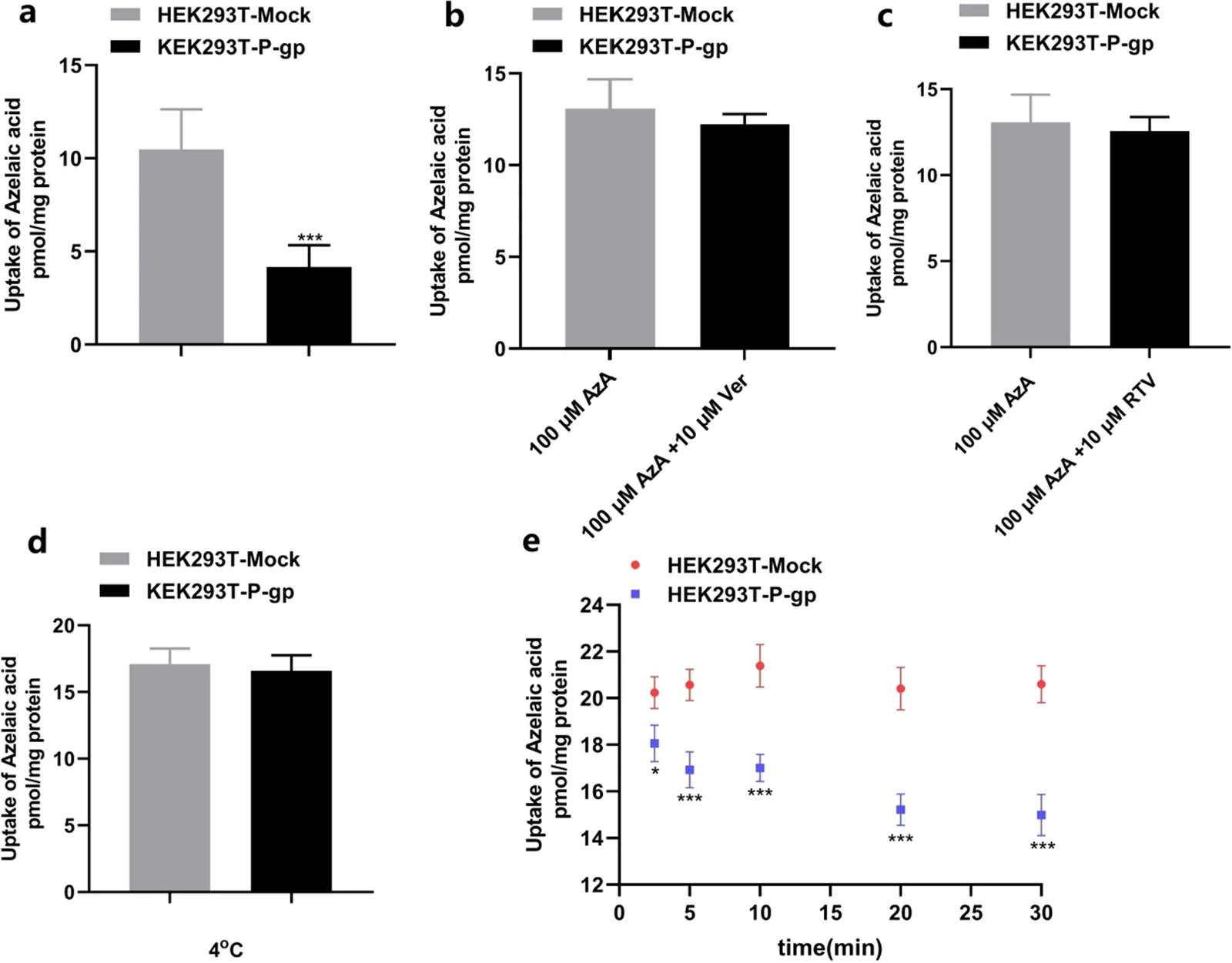
After 20 min of cellular uptake, AzA (100 μM) was taken up by P-gp over-expressing cells (**a**), and the transport activity was inhibited by Ver (10 μM) (**b**), RTV (10 μM) (**c**) and at 4 °C (**d**). Time-dependent uptake was quantified by measuring the amount of AzA (100 μM) in the cells after different time (2.5, 5, 10, 20, 30 min) (**e**). Data are expressed as mean ± SD. (n = 6). *P < 0.05, ***P < 0.001 vs. MDCK-Mock or HEK293T-Mock, respectively. *AzA* Azelaic acid, *Ver* verapamil, *RTV* ritonavir

After 20 min of cellular uptake, there was no significant increase in uptake of 1-methylnicotinamide, pyridoxine, L-serine-O-sulfate, N-acetylornithine, or N-acetyl-L-phenylalanine in the HEK293T-OATP1B3 cells (Fig. s2). As shown in Fig. 3, the uptake of AzA in HEK293T-OATP1B3 cells was significantly increased compared to HEK293T-Mock (*P* < 0.05) (Fig. 3a). AzA was not taken up by MDCK-OATP1B1 cells (Fig. 3b). The transport activity of AzA in HEK293T-OATP1B3 cells was inhibited by RIF (10 μM) and at 4 °C (Fig. 3c and d). Time-dependency experiments demonstrated that at the 5, 10, 20 and 30 min time points uptake of AzA in HEK293T-OATP1B3 cells was significantly higher compared to HEK293T-Mock cells (*P* < 0.05) (Fig. 3e), after which liner uptake time (10 min) was selected and used to measure concentration-dependent uptake in HEK293T-Mock and HEK293T-OATP1B3 cells. In concentration-dependency experiments, the uptake of AzA was significantly higher in HEK293T-OATP1B3 cells compared to HEK293T-Mock cells (Fig. 3f). The Km value of OATP1B3 for AzA was 573.45 μM. The uptake of AzA was significantly decreased in P-gp-overexpressing cells (*P* < 0.01) (Fig. 4a). The transport activity of AzA in HEK293T-P-gp cells was inhibited by Ver (10 μM) (Fig. 4b), ritonavir (10 μM) (Fig. 4c) and at 4 °C (Fig. 4d). Time-dependency uptake demonstrated that at 2.5 min, 5 min, 10 min, 20 min and 30 min (Fig. 4e) point uptake of AzA in HEK293T-P-gp cells was significantly decreased compared to HEK293T-Mock cells.

### Validation of AzA in vivo

**Fig. 5:**
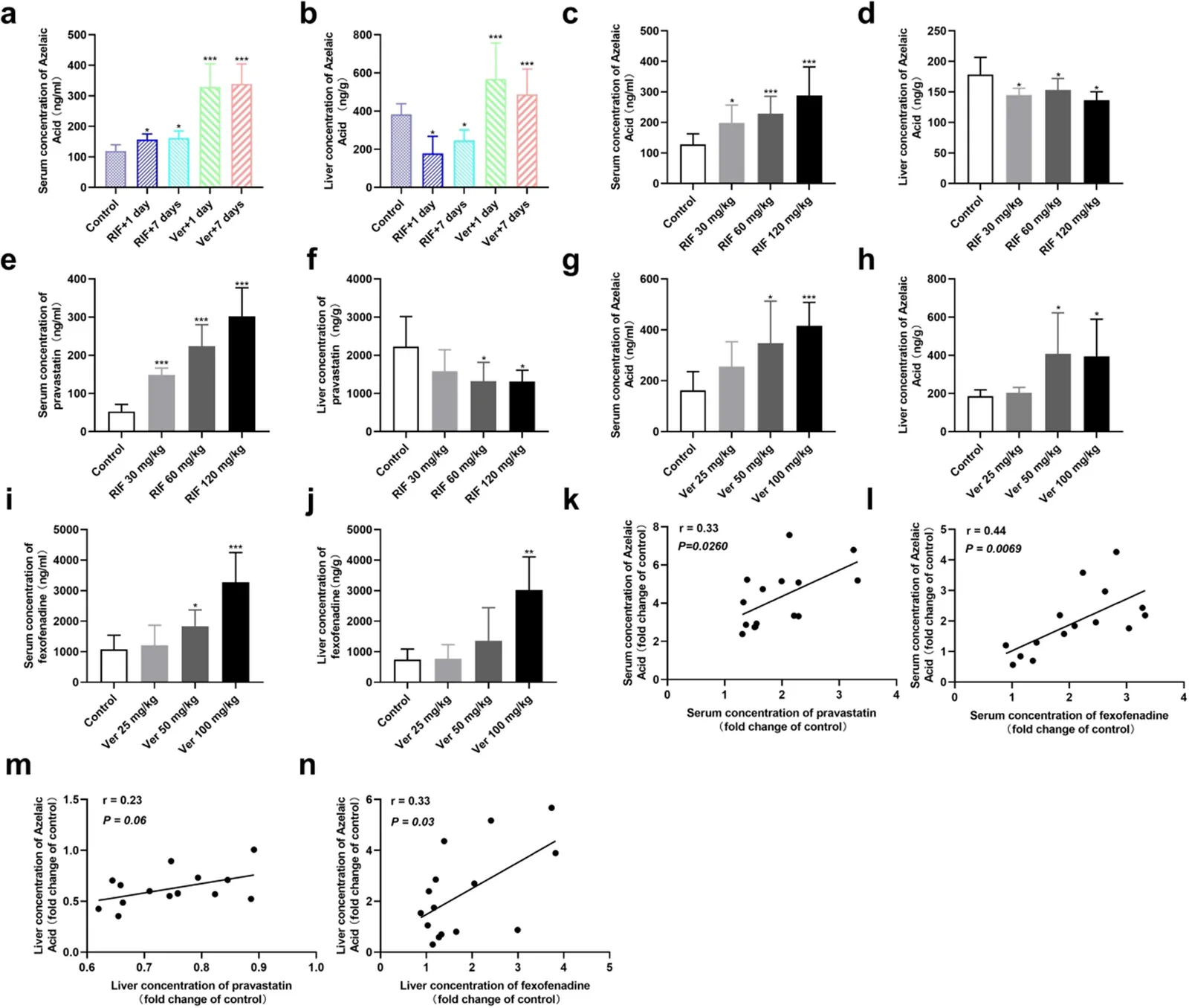
The effect of Ver and RIF on the serum and liver AzA levels in rats. Blood and liver tissue samples were collected 2 h after the last administration; serum (**a**) and liver (**b**) concentration of AzA; and the correlation between the serum concentration of PV, FEX and AzA in rats after a single RIF (30, 60, 120 mg/kg) and Ver dosing (25, 50, 100 mg/kg). Serum (**c** and **g**) and liver (**d** and **h**) concentration of AzA; serum (**e**) and liver (**f**) concentration of PV; serum (**i**) and liver (**j**) concentration of FEX; individual correlation analysis between serum AzA and PV (**k**); individual correlation analysis between serum AzA and FEX (**l**), individual correlation analysis between liver AzA and PV (**m**); individual correlation analysis between serum AzA and FEX (**n**). Data are expressed as mean ± SD, n = 5, *P < 0.05, **P < 0.01, ***P < 0.001 vs. control group, respectively; *Ver* verapamil, *RIF* rifampicin, *AZA* azelaic acid, *PV* pravastatin, *FEX* fexofenadine

To determine if AzA was an endogenous substrate for OATP1B3 and P-gp in vivo, rats were given RIF or Ver once or 7 d consecutively. As shown in Fig. 5a and b, the serum concentration of AzA in RIF-treated rats was higher than that of vehicle-treated rats with single and 7 d treatments (*P* < 0.05). The liver concentration of AzA in RIF-treated rats was lower than that of vehicle-treated rats after single and 7 d treatments (*P* < 0.05). The serum and liver concentration of AzA in Ver-treated rats was higher than that in vehicle-treated rats after both single and 7 d treatments (*P* < 0.05).

### Correlation analysis of the serum concentration of PV, FEX and AzA

We investigated the correlation between the serum concentration of PV, FEX and AzA in rats with the administration of RIF and Ver. Serum levels of AzA and PV were significantly increased (*P* < 0.05) in the RIF (30 mg/kg), RIF (60 mg/kg) and RIF (120 mg/kg) groups (Fig. 5c and e). The hepatic levels of AzA and PV were markedly decreased in RIF (60 mg/kg) and RIF (120 mg/kg) compared to that of the control group (P < 0.05) (Fig. 5 d and f). In RIF (30 mg/kg), the hepatic levels of AzA were markedly decreased (*P* < 0.05), and that of PV was decreased, although not significantly. The serum and hepatic levels of FEX and AzA were markedly increased in the Ver (50 mg/kg) and Ver (100 mg/kg) groups compared to that of the control group (*P* < 0.05) (Fig. 5g–j). There was no significant change in the serum and hepatic levels of FEX and AzA in 25 mg/kg Ver-treated rats. The serum concentration of AzA was highly correlated with the serum level of PV (r = 0.33, *P* < 0.05) (Fig. 5k). Moreover, the serum concentration of AzA was highly correlated with that of FEX (r = 0.44, *P* < 0.01) (Fig. 5l). The liver concentration of AzA was not or only slightly correlated with the liver level of PV (r = 0.23, *P* < 0.06) (Fig. 5m). The liver concentration of AzA was highly correlated with that of FEX (r = 0.33, *P* < 0.03) (Fig. 5n).

### Effect of liver injury on the AzA level in vivo

**Fig. 6:**
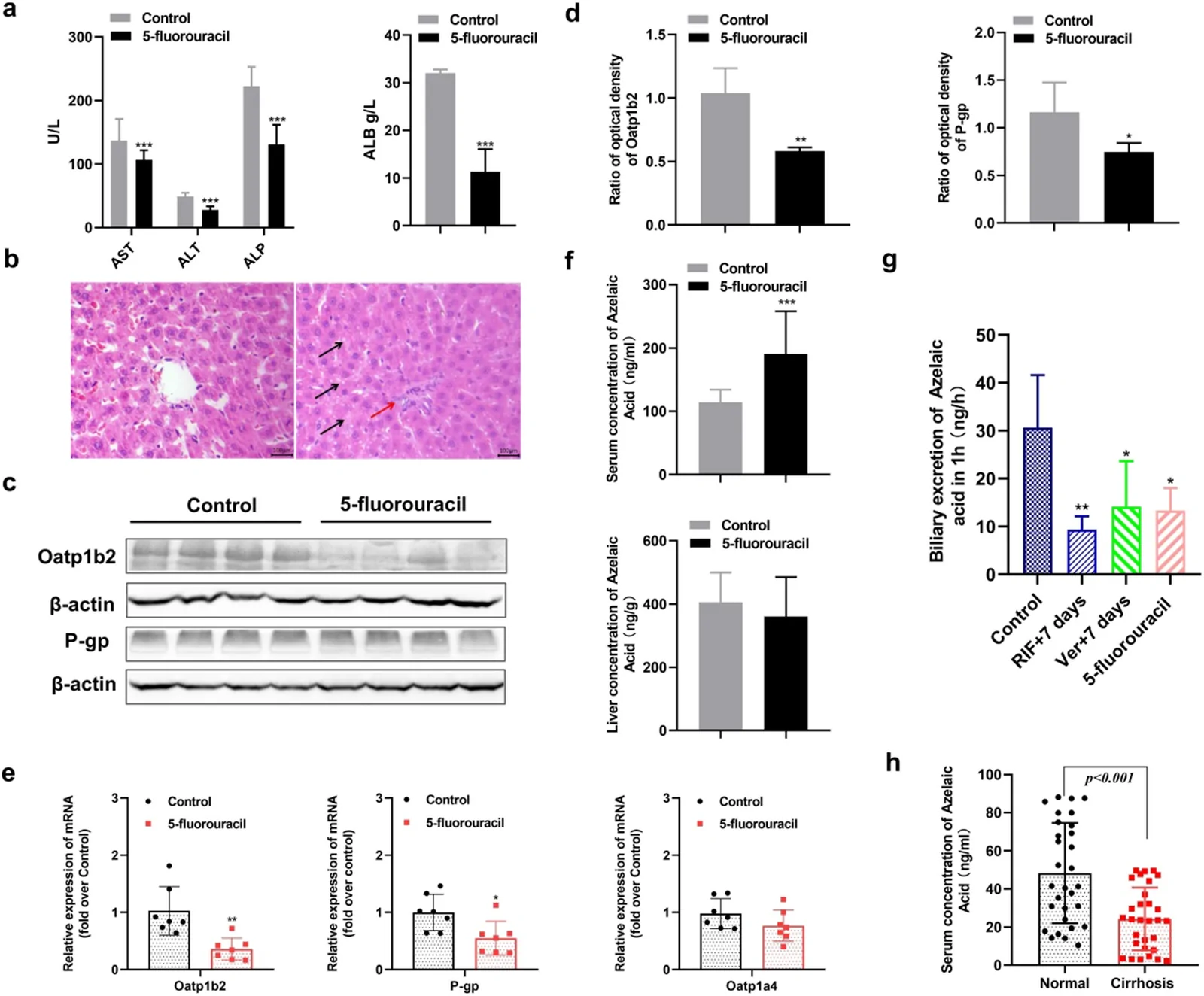
The effect of liver injury on the serum level of AzA after 7 d of 5-fluorouracil dosing. Serum levels of AST, ALT, ALP and ALB (**a**, n = 8); photomicrography of liver sections (**b**, at × 400); the expression of Oatp1b2 and P-gp in the liver (**c** and **d**, n = 4); the mRNA expression of Oatp1b2, P-gp and Oatp1a4 in the liver (**e**, n = 7); serum and liver concentration of AzA (**f**, n = 8); biliary excretion of AzA in rats (**g**, n = 6) and the serum level of AzA in normal subjects and patients with cirrhosis (**h**, n = 30). Data are expressed as mean ± SD, *P < 0.05, **P < 0.01, ***P < 0.001 vs. control or normal group

As shown in Fig. 6, in the 5-fluorouracil-treated group focal steatosis, serum AST, ALT, ALP and ALB levels were markedly decreased in rats treated with 5-fluorouracil (20 mg/kg) (*P* < 0.001) (Fig. 6a), inflammatory cell infiltration and hepatocellular damage were observed (Fig. 6b), suggesting liver injury. The expression of Oatp1b2 and P-gp was significantly decreased in the liver after 5-fluorouracil-induced liver injury (*P* < 0.05) (Fig. 6c and 6d). The relative mRNA level of Oatp1b2 and P-gp was significantly decreased in the liver after 5-fluorouracil-induced liver injury (*P* < 0.05) (Fig. 6e). And there was no significant change in the relative mRNA level of Oatp1a4 in liver (*P* < 0.05) (Fig. 6e). Compared to the control group, the level of AzA was significantly increased in the serum (*P* < 0.05), and there was no significant change in the liver of AzA in the 5-fluorouracil group (Fig. 6f). To determine the influence of transporter inhibitors and liver injury on the biliary excretion of AzA, rats were given RIF, Ver and 5-fluorouracil respectively for 7 d consecutively. As shown in Fig. 6g, the biliary excretion of AzA in RIF-, Ver- and 5-fluorouracil-treated rats was significantly decreased than that of vehicle-treated rats (*P* < 0.05). Moreover, the serum level of AzA was significantly decreased in cirrhosis patients compared to normal subjects (*P* < 0.05) (Fig. 6h).

## Discussion

The organic anion-transporting polypeptide 1b family (OATP1B3) is liver-specific (Csanaky et al. 2011). OATP1B3 is an uptake protein responsible for the uptake of substances from the extracellular space into cells, while P-gp mainly mediates the export of substrates out of cells in an energy-dependent transport. P-gp can be a limiting factor for drug bioavailability (Fromm 2004). OATP1B3 has an overlapping substrate spectrum with P-gp, and the altered function of OATP1B3-P-gp results not only in changes in blood-drug concentrations but also intrahepatic drug concentrations. Therefore, accurate prediction of OATP1B3-P-gp function is important for predicting the pharmacokinetic properties of drug candidates which were vectorially transported by OATP1B3 and P-gp. Endogenous biomarkers have emerged as an approach for drug transporter phenotyping.

In vitro, cell models are recommended as tools to find substrates for transporters. The most commonly used cell lines for uptake studies are HEK293T and MDCK (Hu et al. 2018). In the present study, lentivirus was used to carry the gene for SLCO1B1, SLCO1B3 and ABCB1 into HEK293T and MDCK cells, respectively. The results of WB, LSCM and qPCR indicated that higher OATP1B1, OATP1B3 and P-gp expression was present in MDCK-OATP1B1, HEK293T-OATP1B3 and HEK293T-P-gp cells, respectively than in corresponding HEK293T/MDCK-Mock cells. The transport activity analysis showed that the uptake of PV in the MDCK-OATP1B1 and HEK293T-OATP1B3 cells was significantly increased compared to the corresponding HEK293T/MDCK-Mock cells. The amount of FEX in the HEK293T-P-gp cells was significantly decreased compared to HEK293T-Mock cells. These results demonstrate that stably transfected cells lines were established. Therefore, these cell lines can be used to study OATP1B1, OATP1B3 and P-gp-mediated uptake of substrates into these cells. Metabolomic analysis of biological samples of subjects or animals after the administration of transporter inhibitors or transporter gene knockouts is commonly used for discovering endogenous markers. There are many difficulties in the implementation of clinical trials due to the difficulty of finding suitable biomarkers. Additionally, there are differences in the endogenous compounds between animals and humans. These limit the ability to find suitable biomarkers. In this work, metabolomics with serum pharmacological analysis was used to discover endogenous biomarkers. The use of Krebs–Henseleit buffer containing the dry serum powder sources (equivalent to the serum concentration in humans) of cirrhosis patients as endogenous substrate to identify endogenous biomarkers for the uptake studies in HEK293T-OATP1B3 cells is a highlight of this study. With untargeted metabolomic analysis of cell lysates, a total of sixty-two significant increased metabolites were identified. Six compounds were screened for further study as they are poorly or non-metabolized by liver metabolizing enzymes and have a high VIP value. This eliminates the effect of substrate metabolism in vivo levels. Only AzA was an endogenous substrate of OATP1B3 and P-gp but not OATP1B1. The uptake of AzA by OATP1B3 and P-gp also can be inhibited by the compounds RIF and Ver, respectively, or at 4℃ in vitro. These inhibitors of OATP1B3 and P-gp were administered to rats once or for 7 consecutive days to confirm that AzA is an endogenous substrate for OATP1B3 and P-gp. The results showed that the serum concentration of AzA in RIF-treated and Ver-treated rats was significantly increased both after single and 7 d treatments. A single treatment of inhibitors was to investigate the competitive inhibition of the inhibitor. Multiple doses of inhibitor were to investigate the induction and competitive inhibition of inhibitors. The effects on serum and liver levels of AZA after single and multiple doses of the inhibitors were consistent. It was reported that multiple doses of RIF induced liver enzymes, but inhibited OATP (Zhang et al. 2022; Zheng et al. 2009). Our results suggest that changes in AZA in serum and liver after multiple doses of RIF may be mainly induced by the inhibition of OATP. Moreover, there is a good correlation between the serum concentration of AzA and the probe drugs when rats were treated with inhibitors of OATP1B3 and P-gp in a dose-dependent manner. Results show that the serum concentration of AzA is highly correlated with the plasma level of PV. The serum concentration of AzA is highly correlated with the serum level of FEX. Besides, the liver concentration of AzA was not or only slightly correlated with the liver level of PV, which needed to be examined with further sample size expansion. The liver concentration of AzA was highly correlated with that of FEX. These results indicate that AzA is an endogenous substrate of OATP1B3 and P-gp. Besides, multidrug resistance-associated protein 2 (MRP2), bile salt export pump (BSEP) and breast cancer resistance protein (BCRP) may also play a role in the biliary clearance of some endogenous compounds or drugs. There are no studies related to the transport of AzA by MRP2, BSEP or BCRP. Whether AzA was a substrate of MRP2, BSEP and BCRP still needs further study.

AzA (molecular weight 188.28 Daltons) is a straight, saturated, medium-chain dicarboxylic acid with nine carbon atoms (Mingrone et al. 1989). Serum metabolomics identified AzA as a significantly increased metabolite after 2,3,7,8-tetrachlorodibenzo-p-dioxin-induced oxidative stress in the liver (Matsubara et al. 2012). This was suggested to be an early indicator of liver damage that was related to mitochondrial oxidative stress. The effect of liver injury on the serum level of AzA was investigated in the present study. After 5-fluorouracil-induced liver injury, the level of AzA was significantly increased in the serum, and there was no significant change in the liver concentration of AzA. Oatp1b2 (Slco1b2) has been identified in rats and mice and is thought to be the closest ortholog of human OATP1B3 (Cattori et al. 2000; Choudhuri et al. 2000; Ogura et al. 2000). Some studies showed that OATP1B3 probably corresponds functionally to Oatp1a4 in rat liver (Lee et al. 2017; Nakakariya et al. 2008). The relative mRNA level and expression of Oatp1b2 and P-gp were analyzed in rat liver. The relative mRNA level of Oatp1a4 was also analyzed. This study demonstrated that the significant increase in the AzA level in serum may be induced by decreased expression of Oatp1b2. A decrease in the expression of Oatp1b2 and P-gp led to no significant change in the liver concentration of AzA. Furthermore, when rats were treated with RIF, Ver and 5-fluorouracil respectively, there was a significant decrease in biliary excretion of AzA. This further indicated that AzA was directional transported (OATP1B3-P-gp) from the blood to the bile. The serum concentration of AzA was significantly decreased in cirrhosis patients compared to normal subjects. It has been reported that liver disease increases in P-gp, while OATP1B3 remains unchanged (Drozdzik et al. 2020). The alterations in OATP1B3-P-gp in the liver of cirrhosis patients might explain the significant decrease in AzA in serum. However, clinical trials are further needed.

In conclusion, we established stably-transfected MDCK or HEK293T cells that overexpress human hepatic proteins OATP1B1, OATP1B3 and P-gp. We highlight the importance of using metabolomics with serum pharmacology to identify appropriate endogenous biomarkers for the assessment of transporter function. AzA was found to be an endogenous substrate of OATP1B3 and P-gp. AzA may serve as a potential endogenous biomarker for the assessment of the function of OATP1B3-P-gp for the prediction of changes in the pharmacokinetics of drugs transported by OATP1B3 -P-gp in liver disease states. Further studies still are needed.

## Supplementary Information

Below is the link to the electronic supplementary material.

Supplementary file1 (DOCX 299 KB)

## Data Availability

The data that support the findings of this study are available on request from the corresponding author, Xin-an Wu, upon reasonable request.
