# Supplementary material for: Identification and characterization of endogenous biomarkers for hepatic vectorial transport (OATP1B3-P-gp) function using metabolomics with serum pharmacology
Source: Amino Acids. 2024 Feb 6;56(1):11. doi: 10.1007/s00726-023-03363-5 (PMC10847190; doi:10.1007/s00726-023-03363-5)
Supplement: Supplementary file 1 — Supplementary file1 (DOCX 299 KB) [file 726_2023_3363_MOESM1_ESM.docx]

**Table s1** Sequences for primers for SLCO1B1, SLCO1B3, ABCB1, Slco1b2, Mdr1a and corresponding ACTB.

| Protein code | Sequences for primers |  |
| --- | --- | --- |
| SLCO1B1 | Forward | 5′-TCACTGTCTTTGCATGTGCTG-3′ |
|  | Reverse | 5′-TGACCATACTGTTGCTCTACGT-3′ |
| ACTB | Forward | 5′-CACGGTGCCCATCTATGAGG-3′ |
|  | Reverse | 5′-CCATCTCCTGCTCGAAGTCC-3′ |
| SLCO1B3 | Forward | 5′-AAGGGTCTACTTGGGCTTATCT-3′ |
|  | Reverse | 5′-CAGCAGCATTGTCTTGCATG-3′ |
| ACTB | Forward | 5′-GCGTGACATTAAGGAGAAGC-3′ |
|  | Reverse | 5′-CCACGTCACACTTCATGATGG-3′ |
| ABCB1 | Forward | 5′-GCTCGTGCCCTTGTTAGAC-3′ |
|  | Reverse | 5′-GTGCCATGCTCCTTGACTC-3′ |
| ACTB | Forward | 5′-GCGTGACATTAAGGAGAAGC-3′ |
|  | Reverse | 5′-CCACGTCACACTTCATGATGG-3′ |
| Slco1b2 | Forward | 5′- GGACAGCATCTCAGGCCAAC -3′ |
|  | Reverse | 5′- CACTGGGTTCATTTTGGCGAT -3′ |
| ACTB | Forward | 5′-GTCGTACCACTGGCATTGTG-3′ |
|  | Reverse | 5′-AGGAAGGAAGGCTGGAAGAG-3′ |
| Mdr1a | Forward | 5′-GCAGGTTGGCTGGACAGATT-3′ |
|  | Reverse | 5′-GGAGCGCAATTCCATGGATA-3′ |
| ACTB | Forward | 5′-GTCGTACCACTGGCATTGTG-3′ |
|  | Reverse | 5′-AGGAAGGAAGGCTGGAAGAG-3′ |
| Slco1a4 | Forward | 5′-AGGACTTTTGCTGCTCTGACT-3′ |
|  | Reverse | 5′- GCAGAAGTTTCCTTTCCATTTATGA-3′ |
| ACTB | Forward | 5′-GTCGTACCACTGGCATTGTG-3′ |
|  | Reverse | 5′-AGGAAGGAAGGCTGGAAGAG-3′ |


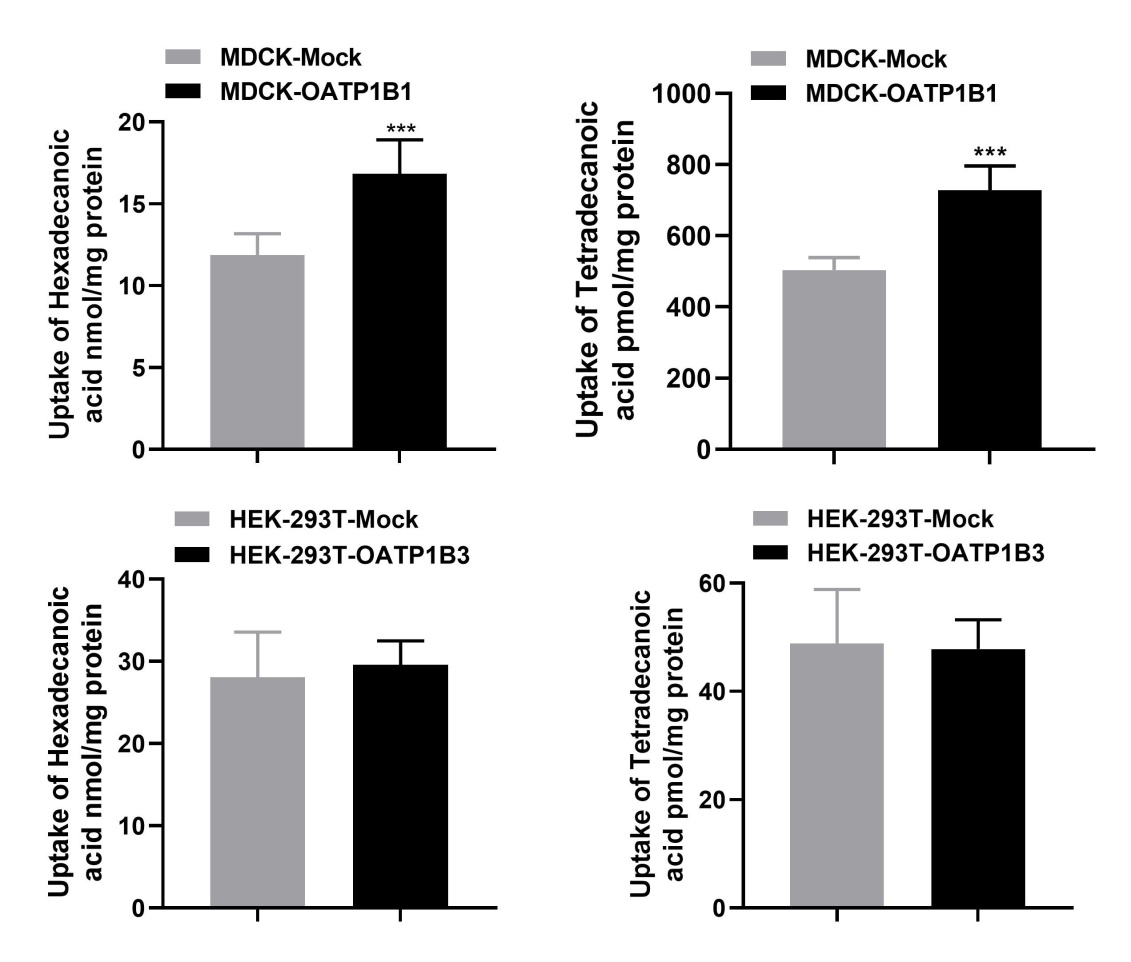


**Fig. s1** After 20 minutes of cellular uptake, tetradecanedioate (10 μM) and hexadecanedioate (10 μM) were taken up by MDCK-OATP1B1 but not by HEK293T-OATP1B3. Data are shown as mean ± SD. (n=6); ***p <0.001 vs. MDCK-Mock or HEK293T-Mock, respectively.


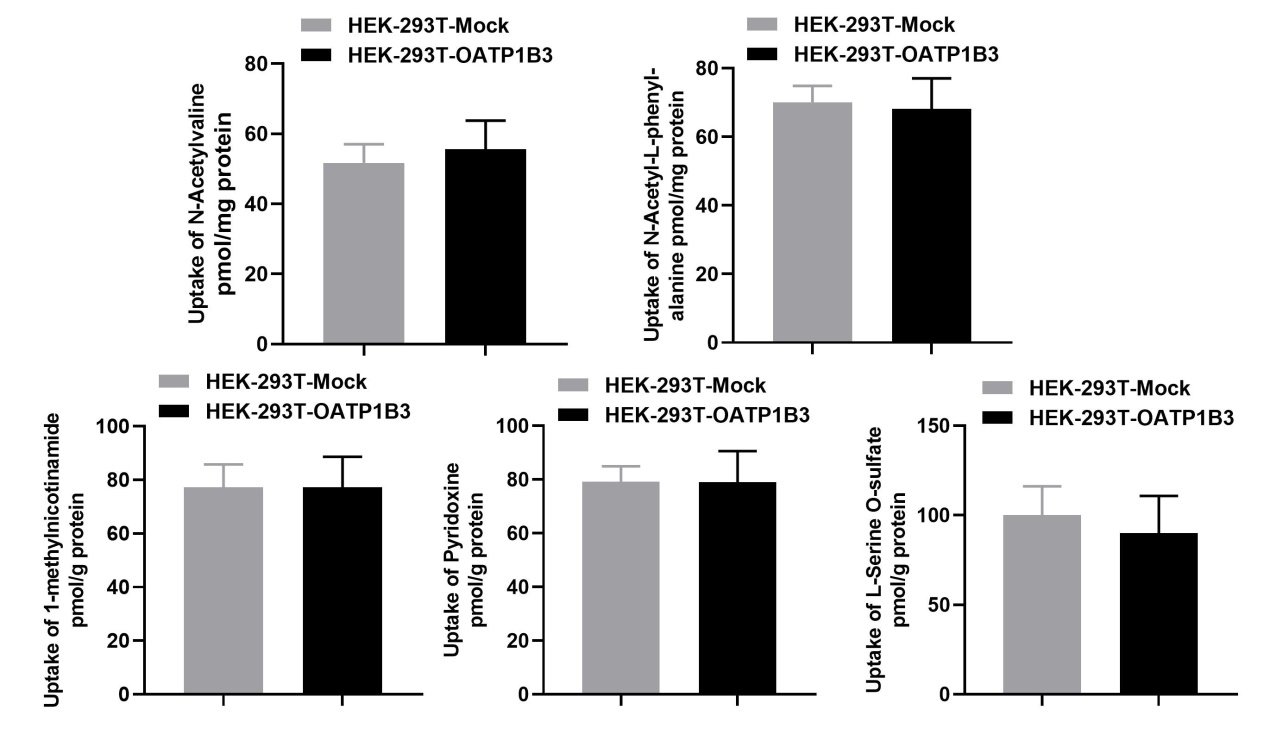


**Fig. s 2** After 20 minutes of cellular uptake, 1-Methylnicotinamide (100 μM), pyridoxine (100 μM), L-serine-O-sulfate (100 μM), N-acetylornithine (100 μM), and N-acetyl-L-phenylalanine (100 μM) were not up taken by HEK293T-OATP1B3. Data are expressed as mean ± SD. (n=6) .
